# Drosophila dyskerin is required for somatic stem cell homeostasis

**DOI:** 10.1038/s41598-017-00446-8

**Published:** 2017-03-23

**Authors:** Rosario Vicidomini, Arianna Petrizzo, Annamaria di Giovanni, Laura Cassese, Antonella Anna Lombardi, Caterina Pragliola, Maria Furia

**Affiliations:** 1 Department of Biology, University of Naples “Federico II”, Complesso Universitario Monte Santangelo, via Cinthia, 80126 Napoli, Italy; 2 NICHD (National Institute of Child Health and Human Development), Section on Metabolic Regulation - NIH- 35 Convent DR, Bethesda, MD 20814 USA

## Abstract

*Drosophila* represents an excellent model to dissect the roles played by the evolutionary conserved family of eukaryotic dyskerins. These multifunctional proteins are involved in the formation of H/ACA snoRNP and telomerase complexes, both involved in essential cellular tasks. Since fly telomere integrity is guaranteed by a different mechanism, we used this organism to investigate the specific role played by dyskerin in somatic stem cell maintenance. To this aim, we focussed on *Drosophila* midgut, a hierarchically organized and well characterized model for stemness analysis. Surprisingly, the ubiquitous loss of the protein uniquely affects the formation of the larval stem cell niches, without altering other midgut cell types. The number of adult midgut precursor stem cells is dramatically reduced, and this effect is not caused by premature differentiation and is cell-autonomous. Moreover, a few dispersed precursors found in the depleted midguts can maintain stem identity and the ability to divide asymmetrically, nor show cell-growth defects or undergo apoptosis. Instead, their loss is mainly specifically dependent on defective amplification. These studies establish a strict link between dyskerin and somatic stem cell maintenance in a telomerase-lacking organism, indicating that loss of stemness can be regarded as a conserved, telomerase-independent effect of dyskerin dysfunction.

## Introduction

Eukaryotic dyskerins are multifunctional proteins that, within the nucleus, associate with three highly conserved proteins (NOP10, NHP2 and GAR1) and one molecule of H/ACA small nucleolar RNA (snoRNA) to form the H/ACA snoRNP machinery required for RNA pseudouridylation^1^. In this process, dyskerins act as catalytic pseudouridine synthases, directing the isomerization of specific uridines to pseudouridines, while each assembled snoRNA acts as a guide to select the specific site on target RNA^2^. The most common targets of pseudouridylation are rRNAs, although pseudouridylation can also influence folding and activity of tRNAs, snRNAs and also mRNA^3–6^. Mammalian dyskerins, through their ability to bind the telomerase RNA component (TERC), participate also to the formation of the active telomerase holoenzyme that is assembled in the Cajal bodies and preserves telomere integrity^7^. Proper functionality of these ubiquitous proteins is crucial, as testified by the observation that hypomorphic mutations of *DKC1*, the gene encoding human dyskerin^8^, cause X-linked Dyskeratosis Congenita (X-DC). X-DC is a severe multisystemic disorder characterized by early aging, increased susceptibility to cancer and loss of stemness. Beyond its well documented role in ribosome biogenesis and telomerase stability, mammalian dyskerin has recently been identified as co-transcriptional regulator of key pluripotency-related genes critical for self-renewal in embryonic stem cells^9^, further highlighting an essential role as regulator of stem cells maintenance. Given the multiple essential functions played by this highly conserved protein, a crucial issue is to distinguish the effects caused by defects in telomerase function^10, 11^ from those caused by defective pseudouridylation^12–14^. This issue can properly be investigated in animal models, since eukaryotic dyskerins are encoded by a highly preserved gene family, whose members include yeast *Cfb5*, rat *Nap57*, mouse *DKC1*, and *Drosophila mfl*/*Nop60b* ^14^. In this respect, *Drosophila melanogaster*, whose telomere stability is not dependent by a canonical telomerase^15^, provides an excellent model to evaluate the telomerase-independent functions played by dyskerins.

Several years ago we showed that *Drosophila* dyskerin is involved in rRNA processing and pseudouridylation^16^. Hypomorphic mutations of *mfl* gene causes developmental delay, defective maturation of rRNA, small body size, alterations of the abdominal cuticle and reduced fertility, implying a key role in growth and developmental processes. In more recent works, tissue-specific silencing was extensively used to reduce the levels of the protein, and resulted in specific alterations of developmental patterns^17^ and occurrence of localized apoptosis and tissue remodeling^18^. In order to investigate whether also the key function in stemness homeostasis is evolutively conserved, we drawed our attention to *Drosophil*a midgut, that in recent years has emerged as an advantageous and well characterized model system for the study of mechanisms governing somatic stem cell maintenance^19^. By using this valuable system for stemness analysis, we found that Drosophila dyskerin is specifically required for the formation of the larval intestinal stem cell niches, while is dispensable for other midgut cell types. Upon dyskerin depletion, the number of adult midgut precursor stem cells (AMPs) is strongly reduced, and these cells do not aggregate into niches. However, a few precursors that remain dispersed into the depleted midguts do not exhibit evident cell-growth defects, nor prematurely differentiate or die. Moreover, these depleted progenitors still retain the expression of the stemness markers *escargot*, *Delta* and *Arm*/*β-catenin*, thus preserving stem cell identity. Instead, dyskerin expression was found to be specifically required within AMPs during embryonic and larval stages for their proper amplification. Altogether, these data further strengthen the notion that dyskerin depletion triggers context-dependent effects, and reveal a general requirement of eukaryotic dyskerins for the proper maintenance of somatic stem cell pool.

## Results

### *Drosophila* dyskerin is required for the formation of larval midgut stem cell niches

Ubiquitous RNAi-mediated knockdown of Drosophila dyskerin (the MFL protein) causes lethality at the onset of metamorphosis, underlining the crucial role played by this gene on *Drosophila* development^17, 20^. Given that silenced adult flies were not viable, we focussed on larval midguts to check the role specifically played by the protein on the intestinal stem cell lineage. This organ is composed by only three cells types, all maintained by a hierarchically organized stem cell lineage^21, 22^ and distinguishable on the basis of their morphology and on the differential expression of two key regulatory genes: *prospero* (*pros*) and *escargot* (*esg*). The absorptive polytenic enterocytes (ECs; *esg-pros-*) represent the most common cell type and are characterized by large polyploid nuclei^23, 24^. Diploid cells are rarer and dispersed in the epithelium, and include both the *pros*+ enteroendocrine cells (ees) and the *esg*+ *pros-* “Adult Midgut precursor” cells (AMPs). From early larval stages, AMPs increase their number through a series of symmetric divisions and disperse into the midgut epithelium^25, 26^. Subsequently, at the beginning of the third larval stage, each dispersed AMP undergoes an asymmetric division that generates another AMP cell and a so called peripheral cell (PC), in which Notch signaling is specifically activated^27^. The newly formed AMP then undergoes a few rounds of symmetric divisions (from 3 to 5), generating an expanded cluster of AMPs (from 4 to 16 AMPs) closely surrounded by one or two falcet-shaped PCs. This structure, called “imaginal island”^27^, represents the transient larval midgut stem niche^25, 28, 29^.

**Figure 1:**
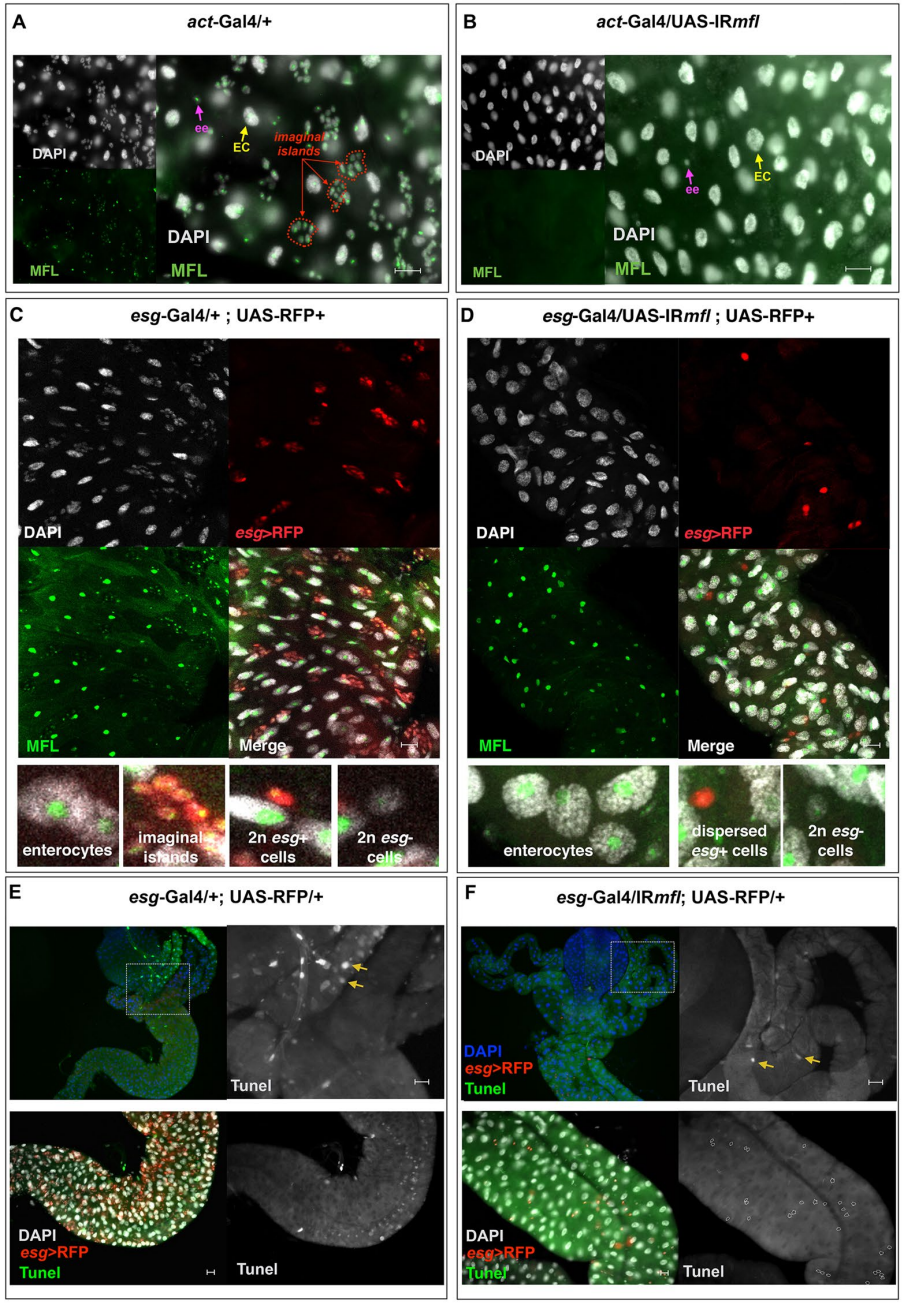
Depletion of Drosophila dyskerin (MFL protein) disrupts the formation of larval midgut stem cell niches. Control (**A**,**C,E**) and silenced (**B**,**D**,**﻿F﻿**) midguts were analyzed in epifluorescence at the third larval stage. DAPI is in grey; anti-MFL antibody in green; *esg*-expression domain is marked in red by an UAS-RFP reporter line. Scale bars: 20 µm. Yellow arrows mark enterocytes (ECs); purple arrows enteroendocrine cells (ees,); red arrows stem cell niches (imaginal islands). In (**A**), all nuclei accumulate the MFL protein. In (**B**), ubiquitous gene silencing drastically abolishes MFL accumulation in all cell types, but specifically disrupts only formation of imaginal islands. In (**D**), MFL depletion was restricted to *esg*+ cells and resulted in the same outcome. (**E**,**F**) TUNEL staining of control (**E**) and silenced (**F**) midguts. DAPI is in green or grey; TUNEL in green or gray; *esg*-expression domain is marked in red by an UAS-RFP reporter line. Note that in both samples only a very few cells are stained (see yellow arrows); moreover, the rare dispersed *esg*+ cells present in the silenced midgut (encircled in the right inset) remain unstained, indicating that they are not undergoing apoptosis.

As shown in Fig. 1A, the three midgut distinct cell types (ECs, ees, AMPs clustered into islands) can easily be morphologically distinguished and all express the ubiquitary MFL protein which, as previously described for other tissues^16, 30^, concentrates in the nucleoli. Upon ubiquitous silencing (genotype: *act*-Gal4/UAS-*mfl*-RNAi) accumulation of MFL protein was efficiently blocked in each midgut cell types; however, the ubiquitous silencing elicited uniquely a dramatic disappearance of the imaginal islands, which were undetectable even upon late third larval stage (Fig. 1B). Hence, the level of the protein appears to be critical for proper formation of larval midgut stem niches, although dispensable in EC and ee differentiated cell types. To further check this point, we restricted gene silencing to the subpopulation of midgut cells expressing *esg*, a *Drosophila* member of the *Snail*/*Slug* family of transcription factors. In the midgut, *esg* expression is limited to components of the islands (AMPs, PCs) and to a subset of ee cells^26, 29^. Intriguingly, no island was again formed in this silencing condition (genotype: *esg*-Gal4/UAS-IR*mfl*; UAS-RFP/+); furthermore, the number of the *esg*+ cells, marked by RFP, was found dramatically reduced (see below for quantification), although rare and dispersed *esg*+ cells which do not accumulate MFL were still present (Fig. 1C,D).

Since in the wing discs *mfl* silencing causes apoptosis^17, 18^, we wondered if the same occurred to MFL-depleted AMPs, thereby causing their loss. We thus stained control and silenced midguts with Terminal deoxynucleotidyl transferase dUTP nick end labeling (TUNEL) in order to mark DNA fragmentation, a characteristic hallmark of apoptosis. According to a previous report, apoptosis is barely detected in wild-type larval midguts, to increase only at the beginning of metamorphosis^31^. Consistent with this, we found that only a few cells were stained in the control midguts (Fig. 1E). Importantly, the signal did not increase in the silenced midguts, nor marked the rare and dispersed *esg*+ cells (Fig. 1F). In addition, an immunostaining with an antibody against the activated form of Caspase-3 (Cas-3), that efficiently marks dying cells in the silenced wing discs^18^, failed to detect any apoptotic spot in the silenced midguts (Fig. S1). Thus, these findings argued against the possibility that AMPs loss could be due to apoptosis. This conclusion further supports the emerging view that dyskerin depletion can trigger context-dependent effects.

### Disruption of stem cell niches is a cell-autonomous effect of dyskerin depletion in the *esg*+ subpopulation of midgut cells

**Figure 2:**
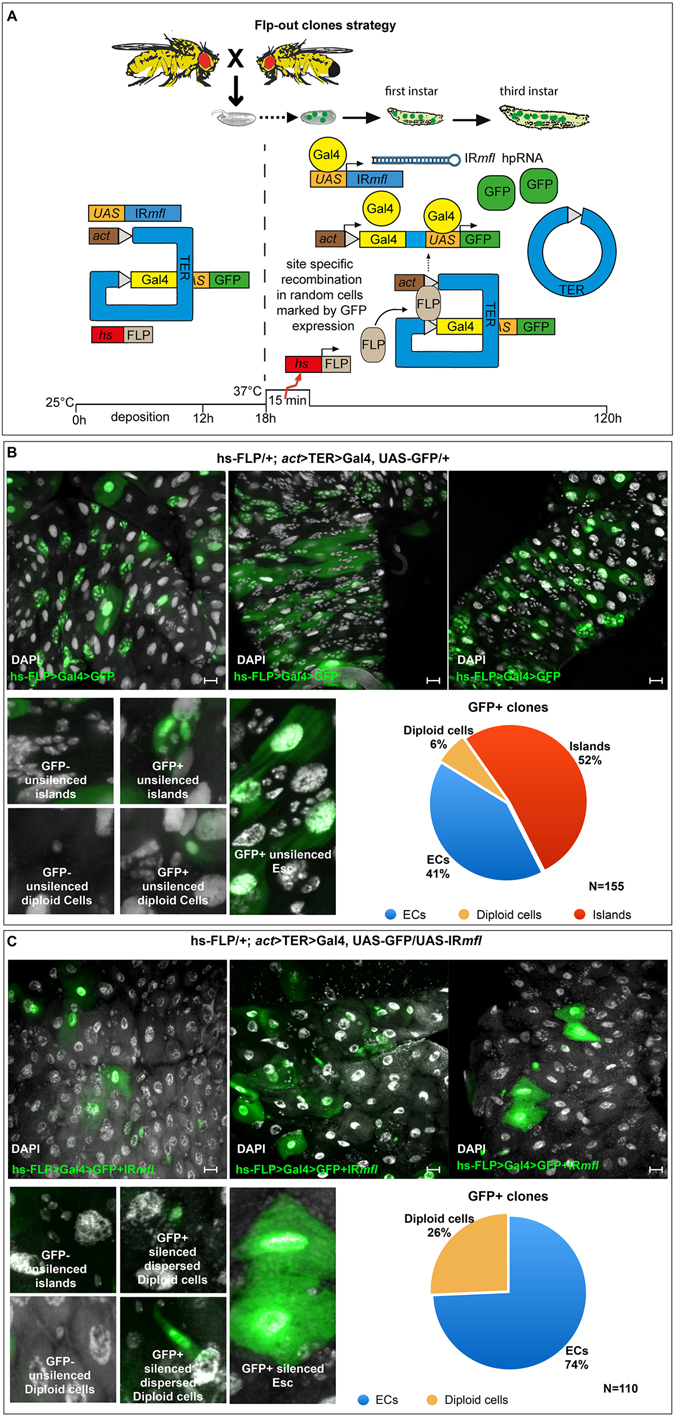
Disruption of the midgut stem cell niches is a cell-autonomous effect. (**A**) Scheme of FLP-OUT clone strategy. Heat-shock-induced expression of Flp fuses *actin* promoter to the Gal4 coding sequence, by removing an internal cassette containing a transcriptional termination site (Stop) flanked by two FRT sites. In clones expressing Gal4, both the UAS-GFP construct (used as marker) and the UAS-IR*mfl* silencing construct were activated. At the third larval stage, midguts were collected, stained with DAPI (in grey) and analyzed in epifluorescence. Different regions of the midgut are shown. Scale bars: 20 µm. (**B**) In control midguts, GFP+ unsilenced clones (N = 155) include imaginal islands (52%), ECs (41%) and unsilenced diploid dispersed cells (6%). (**C**) GFP+ silenced clones (N = 110) are instead formed uniquely by ECs (74%) or by dispersed diploid cells (26%). These clones never include imaginal islands, although these structures are correctly formed in the surrounding GFP- (unsilenced) tissue.

The above results proved that MFL expression is mandatory within the midgut *esg*+ cell population for the formation of the normal pool of the intestinal stem cells. However, *esg* has a dynamic expression profile, with *esg*+ cell population comprising not only the proper imaginal island cells (AMPs and PCs), but also a subgroup of midgut ee cells^26^ and cells from other organs, such as salivary glands, brain and imaginal discs^32^. We confirmed this dynamic expression pattern also at larval stages (see Fig. S2). Consequently, it could not be excluded that the loss of imaginal islands observed in *esg* > *mfl*-RNAi larvae could be a non-autonomous effect triggered by dyskerin depletion in midgut ee cells, or in *esg*+ cells residing outside the midguts. For instance, defective signaling of the insulin-like peptides produced from the brain is able to affect growth and proliferation of AMPs^33^. To firmly assess this point, we performed a mitotic clonal analysis by using the heat shock induced Flp-out technique^34, 35^. In these experiments, outlined in Fig. 2A, Flp-out clones were induced in embryos after they reached the stage of AMPs specification (6 hours AED: After Eggs Deposition^36^), and the midguts were then analyzed at the third larval stage (5 day AED). As expected, in control midguts clones marked by GFP expression included all cell types, mainly the clusters of diploid cells corresponding to imaginal islands and the abundant polyploid enterocytes (ECs) (Fig. 2B). Conversely, in MFL-depleted midguts the GFP positive clones were formed exclusively by ECs or dispersed diploid cells. Imaginal islands were never included in these clones, although they were normally formed in the surrounding tissue (Fig. 2C). This finding clearly indicated that the loss of imaginal islands is a cell-autonomous effect caused dyskerin depletion in midgut *esg*+ cells. Moreover, since Flp-out clones were induced in embryos at a developmental time successive to AMPs specification, it can be concluded that islands loss cannot be ascribed to stem cells specification defects.

### Depletion of dyskerin causes a dramatic reduction of midgut stem cell precursors

Having established that the absence of larval intestinal stem niches caused by MFL depletion in *esg*+ cells is a cell-autonomous phenomenon, we decided to quantify the reduction of the *esg*+ cells. With this aim, we counted them at the third larval stage, when imaginal islands are wholly formed, in 5 control (genotype: *esg*-Gal4/+;UAS-RFP/+) and 5 silenced (genotype: *esg*-GAL4/UAS-IR*mfl*;UAS-RFP/+) midguts.

**Figure 3:**
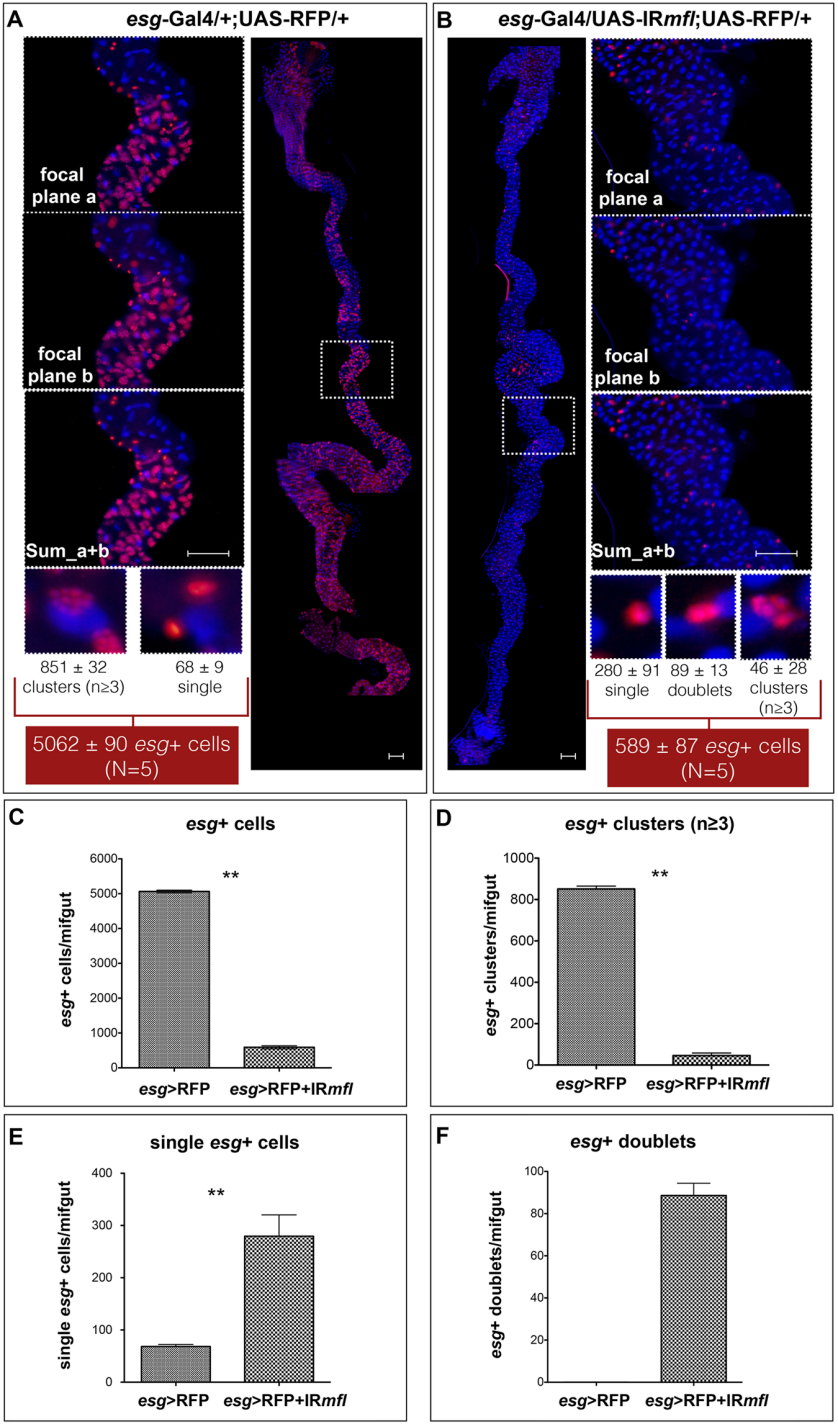
Quantification and organization of *esg*+ cells in whole midguts. Epifluorescence images of control (**A**) and silenced (**B**) midguts at the third larval stage. Scale bars: 100 µm. The white-hatched squares indicate fields whose magnifications are shown aside, as either single focal planes (a,b) and as sum of the two focal planes (a + b). Organization of *esg*+ cells, marked by RFP expression, was analyzed in 5 control and 5 silenced midguts. DAPI is in Blue. Histograms represent the total number: of the *esg*+ cells, regardless the specific kind of organization (**C**); of *esg*+ clusters (**D**); of *esg*+ singlets (**E**); and of *esg*+ doublets (**F**) in control and silenced midguts. Mann-Whitney test was applied for statistical analysis.

The reconstructions of a control (A) and of a silenced midgut (B) are shown for examples in Fig. 3. As it can be noticed, in control midguts the *esg*+ cells appear as clusters of n ≥ 3 cells (imaginal islands), or as single cells (likely enteroendocrine cells, ees). In contrast, in the silenced midguts *esg*+ cells appear mainly as singlets, although a few clusters of 2 cells and rare clusters made up of at least 3 cells were also detected. Furthermore, upon dyskerin depletion we noticed presence of doublets of *esg*+ cells (89 ± 13), usually absent in the control (Fig. 3A,B). We presumed that these cells represented daughter AMPs unable to further proliferate. *esg*+ cells were then counted and the results summarized in Fig. 3. Upon *esg*-GAl4 driven *mfl* silencing, the total number of the *esg*+ cells was found reduced approximately by 88% (Fig. 3C), and the number of the clusters (formed by at least 3 *esg*+ cells) by about 95% (Fig. 3D). Conversely, the number of single dispersed *esg*+ cells increased from 1.3% (68 ± 9) to 47.5% (280 ± 91) (Fig. 3E,F). This increment, statistically significant according to Mann-Whitney test, was compatible either with the persistence of dispersed AMPs within the epithelium or with an increase in the number of *esg*+ cells differentiated in ees.

**Figure 4:**
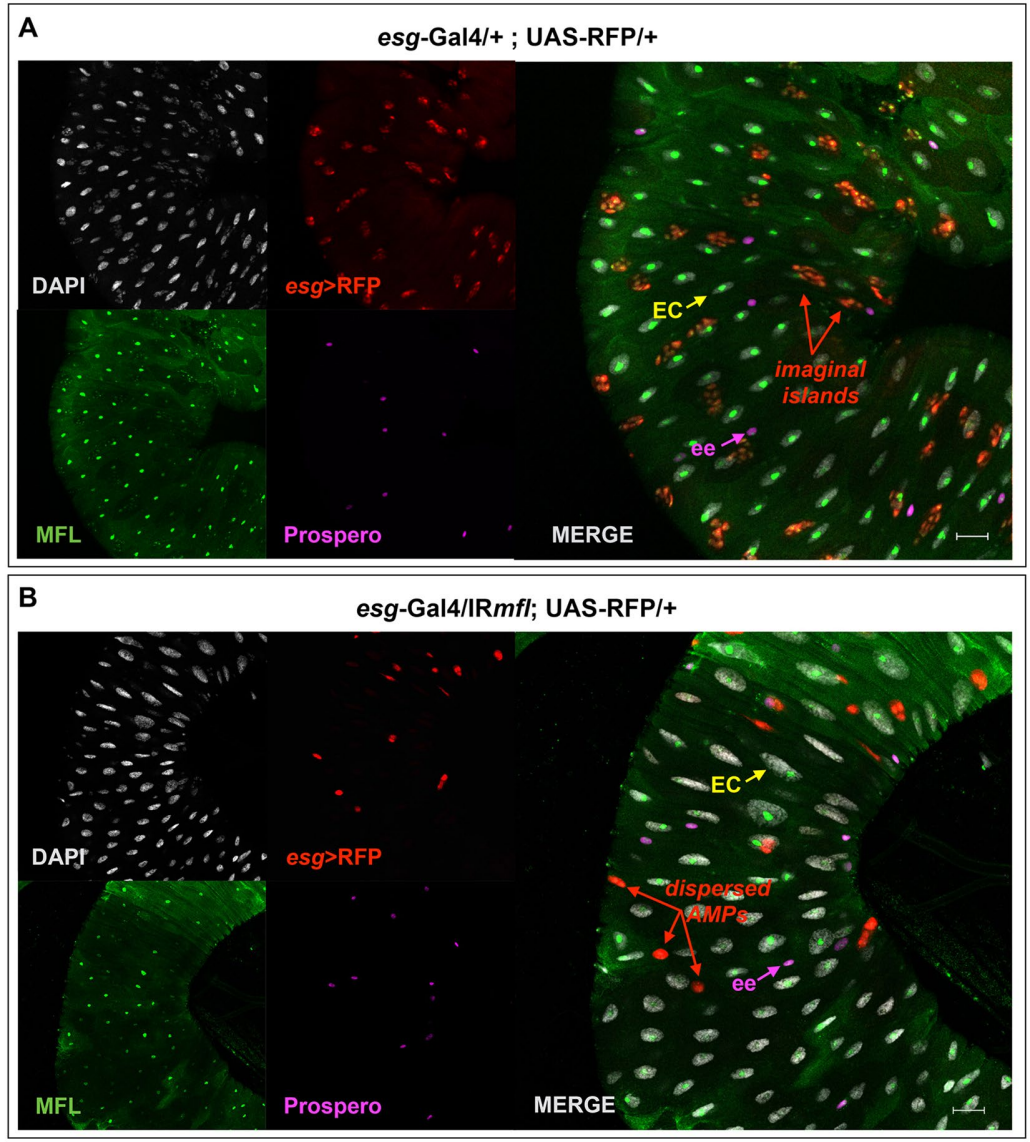
Dyskerin depletion strongly reduces the number of midgut stem cell progenitors. Confocal microscope images of control (**A**) and silenced midgut (**B**) at the third larval stage. DAPI is in gray; *esg*-expression domain is marked in red by the UAS-RFP responder line; staining with anti-MFL is in green; anti-Prospero, which specifically marks ees cells, is in purple. Scale bars: 20 µm. In both (**A** and **B**), purple arrows indicate an ee cell, yellow arrows an enterocyte EC. Red arrows indicate imaginal islands in (**A**), and dispersed *esg*+ *pros*- cells (AMPs and PCs) in (**B**).

In order to clarify whether the excess of *esg*+ dispersed cells found in *esg* > *mfl-RNAi* midguts could originate from a premature differentiation of AMPs in enteroendocrine cells, we monitored the expression of Prospero (Pros) homeoprotein, that represents an ee-specific cell marker^26, 29^. In control midguts (genotype: *esg*-Gal4/+;UAS-RFP/+), the dispersed diploid cells (either *esg*+ or *esg*−) always express Pros, as typical of ee cells (Fig. 4A). Conversely, in the silenced midguts (genotype: *esg*-Gal4/UAS-IR*mfl*; UAS-RFP/+), the majority of the dispersed *esg*+ cells do not express Pros (Fig. 4B); in addition, statistical analysis showed that the number of Pros+ ee cells was substantially unchanged respect to control midguts (Fig. S3). Thus, we concluded that the majority of the dispersed *esg*+ cells are not differentiated ees, but instead dispersed AMPs not clustered into niches.

### Depletion of dyskerin prevents expansion of midgut stem cell precursor from the first amplification phase

**Figure 5:**
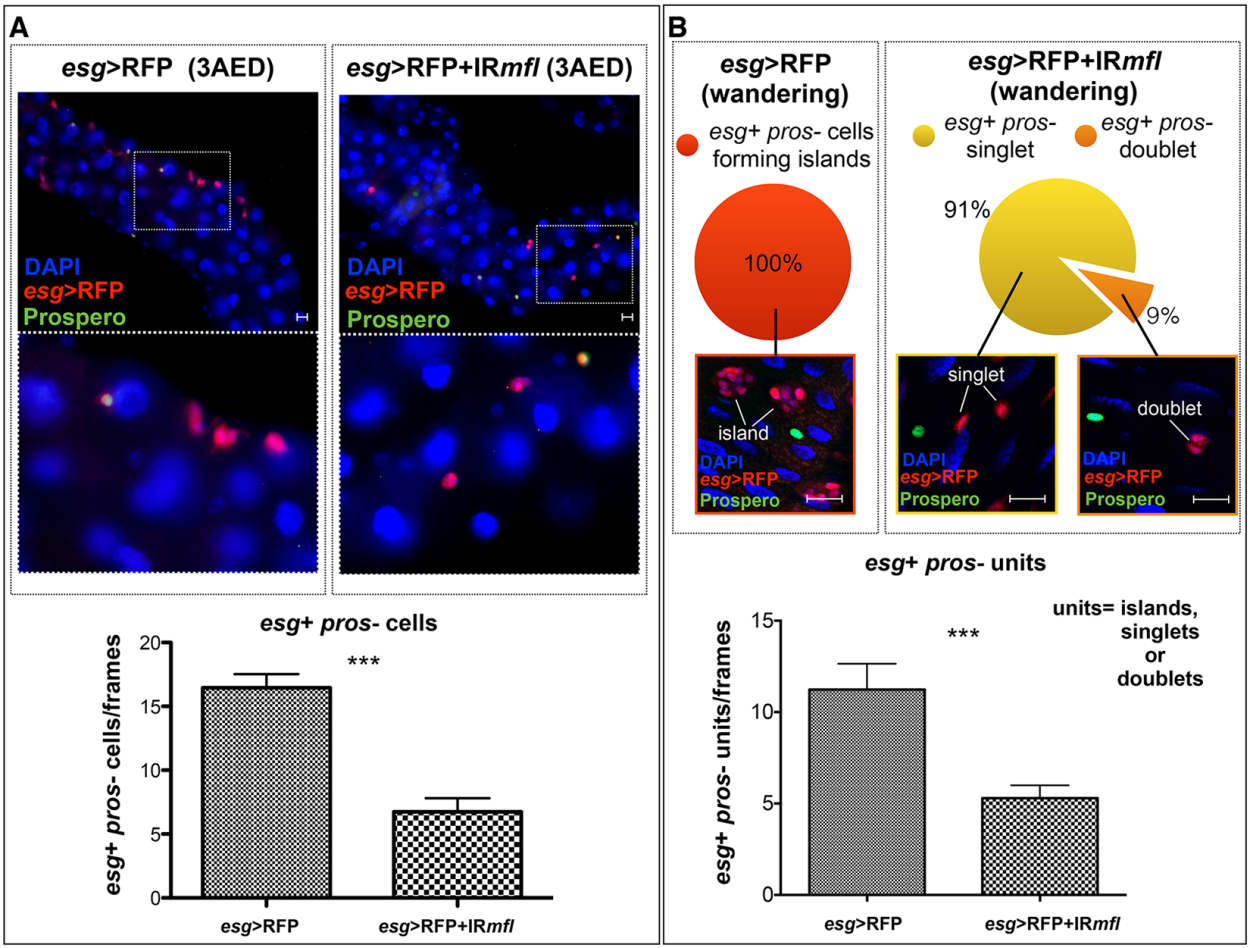
Developmental time-course analysis of stem cell progenitors in dyskerin-depleted midguts. Epifluorescence images of control (*esg*-Gal4/+;UAS-RFP/+) and silenced midguts (*esg*-Gal4/UAS-IR*mfl*; UAS-RFP/+) at 3 days AED (**A**) or at later wandering stage (**B**). DAPI is in blue; *esg*-expression domain in red; staining with anti-Prospero in green. Scale bars: 20 µm. *esg*+ *pros*- cells were counted for frame (at 40X magnification) and the percentage of the different types of organization monitored at the two developmental times; data were subjected to statistical analysis (Mann-Whitney test) and showed by histograms below. (**A**) At 3 days AED, the number of *esg*+ *pros*- cells counted shows a 60% reduction in silenced respect to control midguts. (**B**) At the wandering stage, *esg*+ *pros*- cells are all clustered into imaginal islands (100%) in control midguts, while in silenced midguts 91% of these cells appear as singlets, and 9% as doublets.

As mentioned above, AMP amplification occurs in two phases: a first phase during early larval development (1–3 days AED), when AMPs increase their number and disperse into the epithelium; and a second phase during the mid-third larval stage (4 days AED), when AMPs divide into the imaginal islands^25^. Staining with anti-phospho histone 3 (PH3) antibody fails to directly visualize the proliferation of larval AMPs, although it succesfully marks dividing cells in the adult midgut. According to a previous report^26^, we thus followed the variation in AMPs number during larval development by counting these cells at 3 days AED and at the third instar wandering stage (see Materials and Methods) in both control (genotype: *esg*-Gal4/+;UAS-RFP/+) and silenced (genotype: *esg*-Gal4/UAS-IR*mfl*; UAS-RFP/+) midguts. We identified proper AMPs as *esg*+ *pros-* cells and found their number reduced by approximately 60% at 3 days AED, and by 50% at the later stage (Fig. 5A,B). These reductions -statistically highly significant according to the Mann-Whitney test- pointed out that MFL depletion affects AMPs number primarily during the first amplification phase. Nonetheless, the slight increase in the AMPs number observed at the later stage indicates that a small proportion of *esg*+ *pros-* depleted cells formed in the silenced midguts is still able to undergo a second amplification phase, thereby originating the rare clusters made up of at least 3 *esg*+ cells that can be detected upon silencing.

### The loss of midgut stem cell niches is not due to premature differentiation

Although anti-Pros immunostaining indicated that dyskerin-depleted *esg*+ cells do not differentiate into ees, the possibility of a premature differentiation toward other fates could not be fully excluded. Therefore, we decided to perform a lineage tracing analysis^37^ combining, by genetic crosses, the *esg*-Gal4 driver, the UAS-RFP, UAS-FLP, *ubi* > STOP > GFP/+, and the UAS-IR*mfl* silencing constructs. In the obtained strain, the *esg*-Gal4 driver activates the expression of RFP, UAS-IR*mfl* and Flp specifically in the *esg*+ cells, thus allowing to follow their lineage during larval development. According to this experimental strategy (depicted in Fig. S4), cells which actively express *esg* were marked by RFP, those derived from *esg*+ precursors and continued to express *esg* were marked by both GFP and RFP, and cells derived from *esg*+ precursors which had differentiated, and thus do not express *esg* any more, were marked only by GFP.

**Figure 6:**
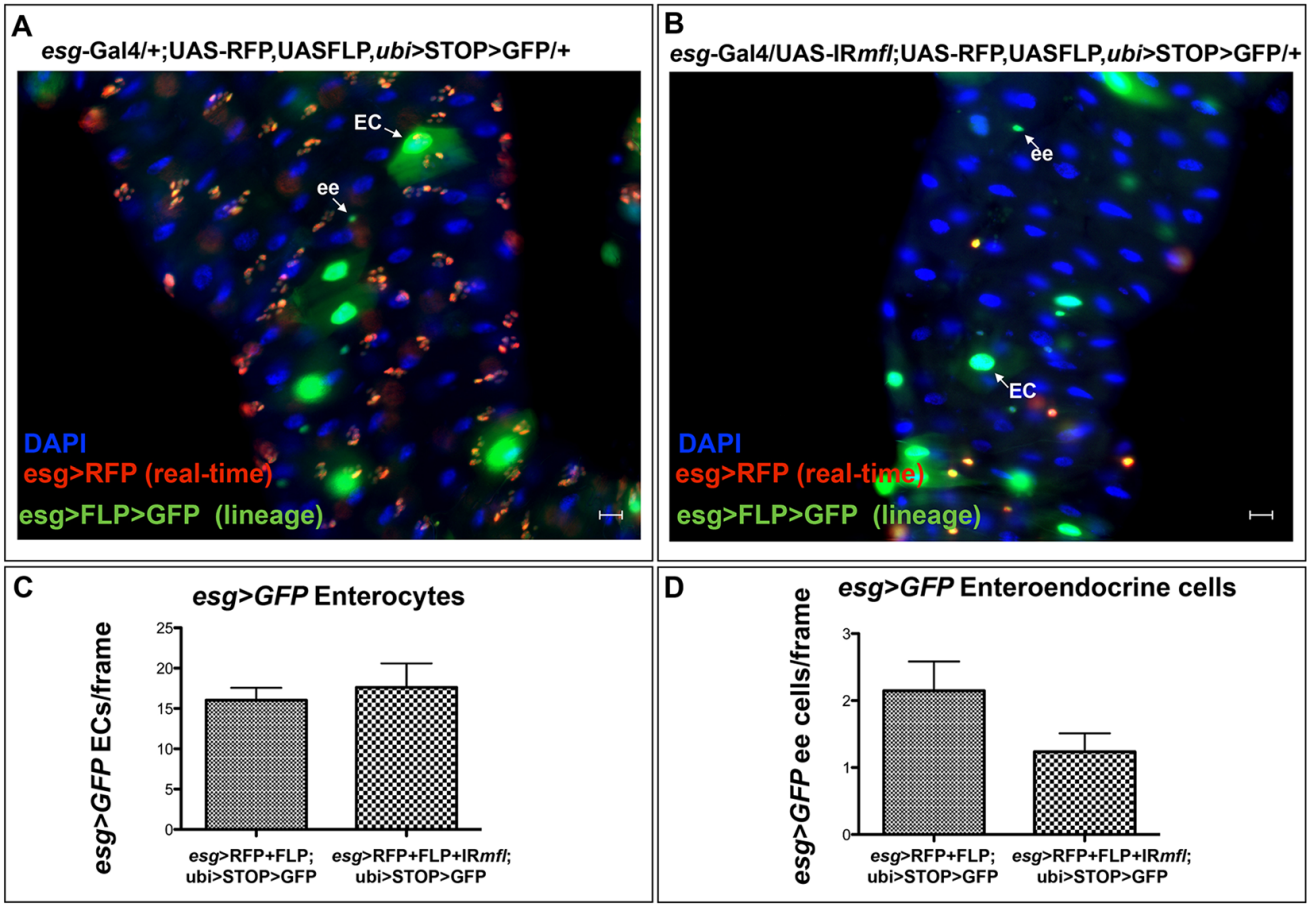
Lineage tracing analysis of midgut *esg*+ cells. Control (**A**) and silenced (**B**) midguts were analyzed in epifluorescence at the third larval stage. DAPI is in blue; GFP in green; *esg*+ cells are marked by RFP in red and appear as clustered into imaginal islands in (**A**) and as dispersed cells in (**B**); white arrows indicate differentiated ECs and ees deriving from *esg*+ precursors. Scale bars: 20 µm. Histograms reporting the number of GFP+ ECs (**C**) and of GFP+ ee cells (**D**) deriving from *esg*+ precursors show that both types of differentiated cells did not significantly increase in silenced respect to control midguts (Mann-Whitney test).

As expected, in control midguts (genotype: *esg*-Gal4/+;UAS-RFP,UAS-FLP,*ubi* > STOP > GFP/+) undifferentiated cells composing the stem niches and deriving from embryonic *esg*+ precursors are marked by both GFP and RFP. Instead, differentiated ECs or ees, which also derive from embryonic *esg*+ precursors, do not express *esg* anymore and are thus marked only by GFP (Fig. 6A). Conversely, in the silenced midguts (genotype: *esg*-Gal4/UAS-IR*mfl*;UAS-RFP,UASFLP, *ubi* > STOP > GFP/+) GFP and RFP expressing cells were represented only by singlets, doublets or by the rare clusters of three cells typically observed in MFL-depleted midguts (Fig. 6B), thus confirming that these cells correspond to AMPs not organized into typical imaginal islands (see above). Remarkably, by comparing the number of GFP+ differentiated cells (ECs and ees) deriving from embryonic *esg*+ precursors in control and silenced midguts, we found that neither the number of ECs or that of ees significantly increased upon MFL depletion (Fig. 6C,D). Altogether, these results ruled out the possibility that reduction in the AMP number could be due to premature differentiation. In addition, our lineage tracing experiments revealed for the first time that only a small number of larval ECs are marked by GFP, and thus derive from *esg*+ cells. Instead, the majority of ECs present in wild-type midguts appears to orginate from embryonic endodermal cells that never express *esg.*

### Dyskerin-depleted midgut stem cell precursors can still express key markers of stemness

**Figure 7:**
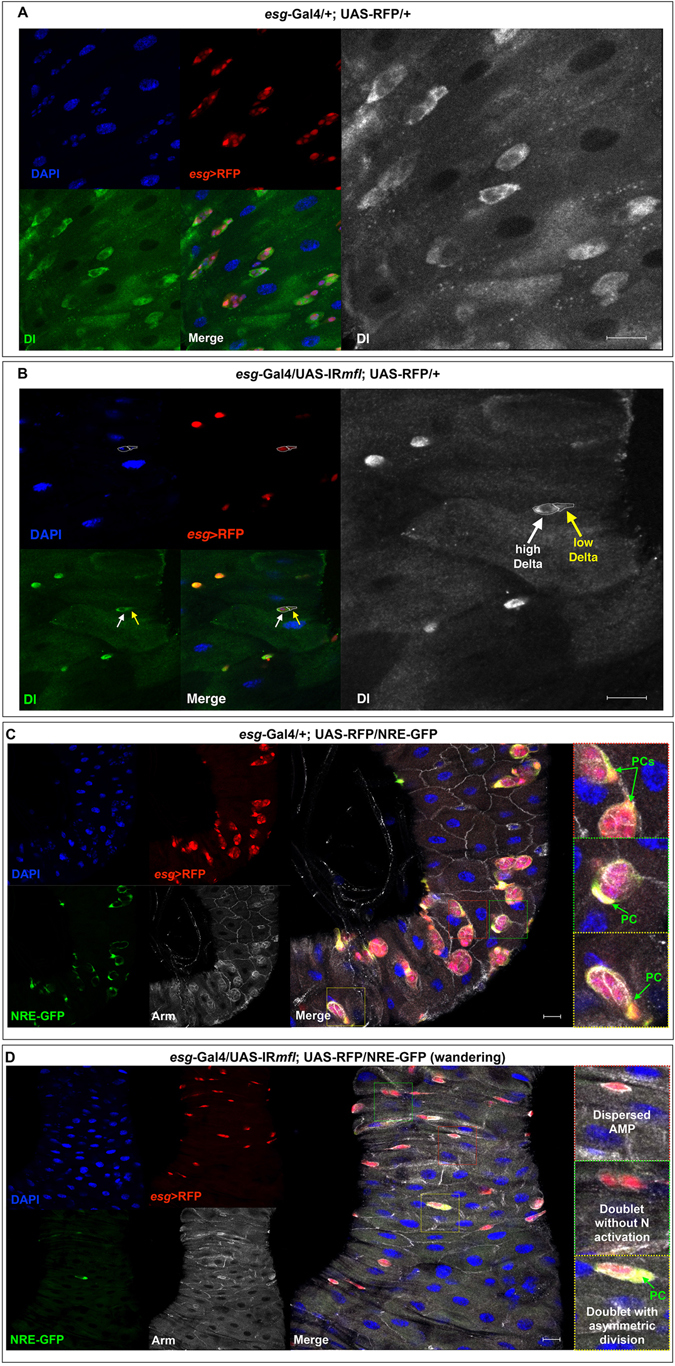
Dyskerin-depleted midgut stem cell precursors can preserve stemness markers. Confocal images of third instar larval midguts were immunostained with anti-Dl (in green; in grey in the enlargements) and anti-Arm (in grey); DAPI is in blue; the *esg* expression domain is marked in red by RFP, and Notch activation is marked in green by the NRE-GFP reporter. Scale bars: 20 µm. In control midguts, Dl localizes in membrane of *esg*+ cells clustered into imaginal islands (**A**); in silenced midguts (**B**), it localizes in membrane of *esg*+ singlets. In addition, Dl is highly expressed in only one member of some doublets (see white arrow), while the other cell shows very poor immunoreactivitiy (yellow arrow). In control midguts (**C**), Arm abundantly accumulates in the adherence junctions of the *esg*+ cells forming the imaginal islands, while Notch activation specifically marks the peripheral cells (PCs) surrounding each island. In silenced midguts (**D**), Arm surrounds *esg*+ MFL-depleted singlets and abundantly accumulates at the adherence junctions of the doublets; in keeping with Dl expression, Notch is found asymmetrically activated in some doublets.

The AMP physiological state is characterized by the expression, in addition to *esg*, of two other key effectors of stemness, such as Notch ligand Delta (Dl)^25, 27, 36^ and Arm/β-catenin^38^. Having established that the stem cells loss observed in the silenced midguts was not caused by massive premature differentiation in ees or ECs, we then asked whether the dyskerin-depleted AMPs dispersed into the epithelium could still preserve their stem cell identity. As expected, in control midguts (genotype: *esg*-Gal4/+;UAS-RFP/+) of third instar larvae, Dl marks the membrane of AMPs aggregated into niches, while Arm accumulates at adherens junctions of those cells composing the imaginal islands (Fig. 7A). In the silenced midguts (genotype: *esg*-Gal4/UAS-IR*mfl*;UAS-RFP/+) the dispersed AMPs also express Dl and Arm, and both proteins appear correctly localized in membranes (Fig. 7B). Moreover, anti-Arm immuno-localization underlined a strict adhesion between the two members of a same *esg*+ doublet, suggesting that both actually derive from a single mother cell. Finally (see Fig. 7B, white and yellow arrows), in some doublets Dl is highly expressed in only one cell, suggesting that the other member of the doublet activated Notch signaling, and thus differentiated as PC^27^. To further address this point, we used the Notch activity reporter NRE-GFP^39^. In control midguts, this reporter specifically marks PCs (Fig. 7C). In the silenced midguts, we noticed that in some *esg*+ doublets it is active in only in one cell (Fig. 7D), supporting the conclusion that some doubles are properly composed by an AMP associated to a PC, as previously suggested by anti-Dl labeling. Thus, even upon MFL depletion some AMPs remain able to divide asymmetrically.

### Dyskerin depletion does not affect the growth ability of midgut stem cell precursors

**Figure 8:**
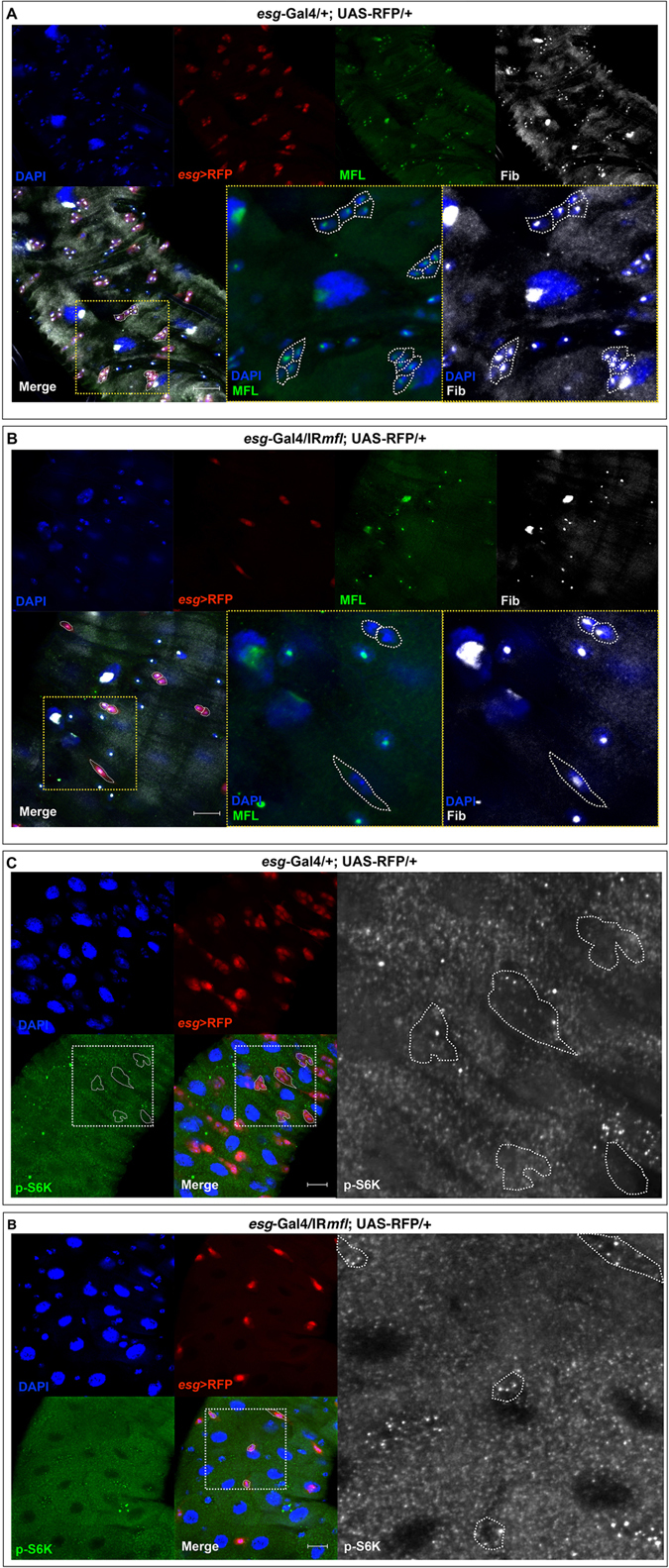
Dyskerin-depleted midgut stem cell precursors do not show growth alterations. Confocal images of third instar larval midguts after immunostaining with anti-MFL, anti-fib and anti- p-S6K. DAPI is in blue; *esg* expression domain is marked in red by RFP; anti-MFL is in green; anti-fib is in grey; anti-p-S6K is in green (and in grey in the enlargements). Scale bars: 20 µm. In either control (**A**) and silenced midguts (**B**), MFL and fib co-localize in the nucleolus, as it can be clearly seen in the enlargements of the yellow-dashed squares, where white dotted lines encircle imaginal islands in (**A**) and dispersed *esg*+ cells (doublets or singlets) in (**B**). In control midguts (**C**), immunostaining with anti-p-S6K reveals a very low accumulation level in cells composing the imaginal islands (encircled by a dotted white line in the enlargement); in the silenced midguts (**D**), similar levels were found in *esg*+ singlets and doublets (encircled by a white dotted line in the enlargement).

Since dyskerins are involved in rRNA processing and ribosome biogenesis, we asked whether the stem cell loss caused by gene silencing could be related to cell growth defects. Cell growth can be evaluated by the levels of specific nucleolar markers^40, 41^, and in fact levels of nucleolar fibrillarin have been shown directly proportional to cell growth ability^42^. We thus firstly evaluated the levels of fibrillarin in the nucleoli of AMPs in either control (genotype: *esg*-Gal4/+;UAS-RFP/+) and silenced (genoptype: *esg*-Gal4/UAS-IR*mfl*; UAS-RFP/+) midguts at the third larval stage. In control midguts, fibrillarin co-localizes with MFL in the nucleoli of all cell types, including those forming the imaginal islands (marked by RFP expression; Fig. 8A). This pattern remains unchanged in the silenced midguts, where fibrillarin accumulation is unaffected also in the nucleoli of dyskerin-depleted *esg*+ cells (doublets or single cells), thus suggesting no evident cell growth impairment (Fig. 8B). Conditions that compromise cell growth are also known to cause the repression of Tor signaling, resulting in low levels of phosphorylation of its downstream effector, the ribosomal protein S6 kinase (S6K)^43^. We then monitored the levels of S6K phosphorylated form (p-S6K) and, as shown in Fig. 8C,D, found no reduction in MFL-depleted *esg*+ cells (genotype: *esg*-Gal4/UAS-IR*mfl;*UAS-RFP) respect to their control (genotype: *esg*-Gal4/+;UAS-RFP/+). Finally, we checked the accumulation levels of phosphorylated form of the translation initiation factor eIF2α (p-eIF2α), which rises in response to growth restraint caused by nutrient deficiencies^44^. No difference between the levels of p-eIF2α levels in the imaginal islands of control midguts (genotype: *esg*-Gal4/+;UAS-RFP/+) and those of AMPs dispersed in the silenced midguts (genotype: *esg*-Gal4/UAS-IR*mfl*;UAS-RFP/+) was noticed, with p-eIF2α levels being very low in both samples (Fig. S5A,B). Altogether, these results lead us to conclude that the defective AMPs amplification observed upon *mfl* silencing is not caused by growth impairments.

## Discussion

Hypomorphic mutations of the *Drosophila* dyskerin gene (*mfl*) have already been shown to critically affect both the maintenance of the male germ-line stem cell lineage^45^ and the early differentiation of germinal stem cells during oogenesis^16, 46^. Although known to be critical in the germ line, as yet the role of *Drosophila* dyskerin in somatic stem cell maintenance has not properly been investigated, even if defective gene expression was shown to cause a plethora of developmental somatic defects^16–18^. By using the hierarchically organized and well characterized cell lineage of the *Drosophila* larval midgut, we demonstrated here that either ubiquitous or *esg*-driven dyskerin depletion specifically abolishes the formation of the intestinal imaginal islands, the transient stem cell niche, without any apparent effect on the other midgut cell types. Moreover, the stem cells precursors are dramatically reduced in their number and appear mostly as single or double dispersed cells, further highlighting that protein accumulation is essential for adequate formation of the pool of intestinal stem cells. We show that these effects are cell-autonomous, thereby implying that dyskerin expression is specifically required within the midgut subpopulation of *esg*+ cells. Remarkably, some depleted precursors can keep the expression of stemness specific markers, such as Armadillo/β-catenin and Delta, and are still able to divide asymmetrically, as testified by the correct activation of Notch signaling in some of the doublets. Several biological phenomena could be compatible with the observed phenotype and were thus considered in the progress of this work. Defective specification, apoptosis, decreased proliferation or premature differentiation of AMPs, could all have a role in the disruption of the stem cells pool. However, mitotic analysis^47^ indicated that stem cells loss is not due to defective specification, while G-trace lineage experiments, which allow to follow the lineage of a specific cell type^37^, ruled out the possibility that stem cell precursors undergone premature differentiation. In addition, the reduction in the precursor number was not due to cell-growth defects or apoptosis. In fact, the expression of the cell-growth markers p-S6K, p-eIF2α and fibrillarin^42–44^, as the number of apoptotic cells, were all essentially unaffected by dyskerin depletion. Rather, a developmental time-course analysis of variation of the AMPs number revealed that the first phase of stem cells amplification is specifically affected.

Altogether, these results point out that eukaryotic dyskerins play a crucial role in somatic stemness homeostasis also in a telomerase-lacking organism as Drosophila, thus suggesting a general requirement for these proteins into stem cell compartments. Indeed, it is reasonable to suppose that dyskerins may regulate evolutively conserved pathways involved into control of stem cells amplification. Although the specific transductional signals remain to be identified, the EGFR/RAS pathway, which has a main role in regulating AMPs amplification in Drosophila larval midgut^25^, may plausibly represent a suitable candidate. However, several additional telomerase-independent roles of dyskerin may be evoked. For example, canonical functions of H/ACA snoRNP complexes, such as those involved in the pseudouridylation machinery, might be needed into stem cell niches at higher levels than in differentiated cells, thereby making theses compartments more sensitive to dyskerin levels. The possibility that ribosome biogenesis and/or ribosome-mediated translational control may differ between stem and differentiated cells also deserves to be considered, and in fact many factors involved in ribosome biogenesis appear to play stem cell-specific roles^48^. Alternatively, dyskerins may regulate essential stemness processes in association with cell-type specific H/ACA ncRNAs^49^. Indeed, this attractive hypothesis is supported by the recent finding that a H/ACA snoRNA specimen (SNORA7A) is highly expressed in undifferentiated umbilical mammalian stem cells and promotes the self‐renewal ability through snoRNP complex^50^.

To this regard, it is also worth noting that in mammalian embryonic stem cells dyskerin acts as co-transcriptional factor of key pluripotency-related genes, such as *Oct4*, *Sox2* and *Nanog* ^9^. This uncanonical role of transcriptional regulator has so far been poorly investigated, and it might possibly be shared by other eukaryotic dyskerins in several stem cell contexts. Worth noting, a specific requirement of dyskerin into somatic stem cell niches is fully consistent with the observation that the mostly affected tissues in X-DC patients are those with high cellular turnover, and that thus need a large pool of somatic stem cells for their renewal. Indeed, the comprehension of the molecular mechanisms at the base of the specific requirement of dyskerins into stem cells could led to better define the etiology of X-DC.

## Materials and Methods

### *Drosophila* strains

Flies were raised on standard *Drosophila* medium at 25 °C. The following strains: #36595 (UAS-IR*mfl*); #4414 (*act*-Gal4); #42732 (*tub*-Gal4); #26816 (*esg*-Gal4); #31417 (UAS-RFP); #28281 (UAS-RFP,UAS-FLP,Ubi > STOP > GFP); #30728 (NRE-GFP) were obtained from Bloomington Drosophila Stock Center at Indiana University (BDSC, Bloomington, IN, USA). The *hs*-Flp; *act* > *TER* > Gal4,UAS-GFP line were kindly provided by D. Grifoni (University of Bologna, Italy). The UAS-IR*mfl*RNAi (v46282) line was obtained from Vienna Drosophila RNAi Center (VDRC, Vienna, Austria).

### Immunofluorescence stainings and microscope analyses

For larval midgut stainings, Drosophila larvae were dissected in PBS, fixed for 2 h in 3,7% PFA, permeabilized in 0,1% PBT (No permeabilization for anti-Dl), blocked in 5% NGS (Normal Goat Serum in PBT, substituted by PBS for anti-Dl) for 1 h at room temperature and incubated overnight at 4 °C in 5% NGS with primary antibody. Tissues were then incubated in 5% NGS with secondary antibody for 1 h at room temperature. Accurate washes in 0,1% PBT (PBS for anti-Dl) were performed following each step. Midguts were then mounted in 70% Glycerol + DAPI for microscope analyses. Antibodies used were: customer rabbit polyclonal antibody against MFL (Sigma-Aldrich Inc., St. Louis, MO, USA; dilution 1:100); mouse monoclonal antibodies against Pros, Dl, Arm (Hybridoma Bank, University of Iowa, Iowa City, IA, USA; dilution. 3–5 μg/ml); rabbit polyclonal antibodies against cleaved Caspase-3, p-S6K, p-eIF2α (Cell Signaling Tech., Danvers, MA, USA; dilution 1:500 anti-Cas3 and anti- p-S6K, 1:50 anti-p-eiF2α); mouse monoclonal antibody against Fib (Abcam; Cambridge, UK, dilution 0.1/100). Fluorescent secondary antibodies were from Jackson Immuno Research (Dianova, Hamburg, Germany) and used at a final dilution of 1:250. Epifluorescent images were obtained with a Nikon Eclipse E1000 microscope (Nikon Instruments Europe, Tokyo, Japan). Confocal images were obtained with Zeiss LSM510 or with Zeiss LSM700 confocal microscopes (Carl Zeiss, Jena, Germany) and analyzed and processed with ImageJ v1.440 software (https://imagej.nih.gov/ij).

### TUNEL staining

Larval midguts were fixed in 4% paraformaldehyde in PBS for 30 min at room temperature. Tissues were permeabilized in 100 mM sodium citrate dissolved in PBTx (PBS plus 0.3% Triton X-100) at room temperature for 30 min, followed by the addition of terminal deoxynucleotidyltransferase mediated dUTP-biotin nick end labeling (TUNEL) mix (APO-BrdU™ TUNEL Assay Kit, Thermo Scientific, Waltham, MA, USA), incubation with Anti-BrdU Alexa Fluor 488 at room temperature for 2 h, and rinsing in PBS. Tissues were then mounted in 70% glycerol + DAPI, and TUNEL-positive cells visualized by epifluorescence microscopy (Nikon Eclipse E1000).

### Quantification of *esg*+ cells in whole midguts

Considering that *mfl* silencing driven by *esg-*Gal4 induces a larval developmental delay of about 2 days, we collected control midguts at 5 days AED and silenced midguts at 7 days AED. The collected midguts were fixed in 3,7% PFA, washed in PBS and mounted in 70% Glycerol + DAPI for epifluorescence analysis. Images were acquired with a 20X objective of Nikon Eclipse E1000 epifluorescence microscope on two focal planes. The two planes were added with STACK > Z Projection ImageJ plug-in (https://imagej.nih.gov/ij) and the stacked images were pieced together with Adobe Photoshop CS5.1 to obtain the reconstruction of entire midguts from the proventriculus to the posterior gut region. The *esg*+ cells marked by RFP fluorescence were classified as single, doublets and clusters of at least 3 cells and counted using the cell counter Image-J plug-in.

### Quantification of midgut *esg*+ *pros*− cells

The *esg*+ cells, marked by RFP fluorescence, were counted in midguts stained with anti-Prospero at 3 days AED and at the third larval stage (5 days AED for control midguts; 7 days AED for silenced midguts). We analyzed frames of midguts (at a magnification of 40X objective of Nikon Eclipse E1000 epifluorescence microscope) and not entire midguts, because the immunostaining method does not allow to keep the tissue intact. The *esg*+ cells were classified as single, doublets and clusters of at least 3 cells and counted using the cell counter Image-J plug-in.

### Quantification of midgut *pros*+ cells

The *esg*+ *pros*+ and *esg*− *pros*+ cells were counted in midguts stained with anti-Prospero at the third larval stage (5 days AED for control midguts; 7 days AED for silenced midguts) upon epifluorescence analysis with a Nikon Eclipse E1000 microscope, at magnification of 40X objective.

### Quantification of midgut GFP+ differentiated cells

The GFP+ enterocytes (ECs) and enteroendocrine cells (ees) in midguts of the correct genotypes were counted at the third larval stage (5 days AED for control midguts; 7 days AED for silenced midguts) in frames at a magnification of 40X objective of Nikon Eclipse E1000 epifluorescence microscope. The cell counter Image-J plug-in was used.

### Flp-out/Gal4 analysis

Drosophila transgenic embryos of correct genotypes were collected after o/n deposition and heat-shocked for 15 min at 37 °C at 18 h AED (i.e. after stage 10 of embryonic development) to induce clones. Larval midguts from heat-shocked animals were dissected at the third larval stage in wandering (120 h AED), and then analyzed.

### Statistical analysis

The Mann-Whitney test for variance analysis was applied. A difference of p < 0.05 was considered statistically significant.

## Electronic supplementary material

Supplementary information.PDF
