# Supplementary material for: Drosophila dyskerin is required for somatic stem cell homeostasis
Source: Sci Rep. 2017 Mar 23;7:347. doi: 10.1038/s41598-017-00446-8 (PMC5428438; doi:10.1038/s41598-017-00446-8)
Supplement: Supplementary file 1 — Supplementary information.PDF [file 41598_2017_446_MOESM1_ESM.pdf]

SUPPLEMENTARY INFORMATION

Drosophila dyskerin is required for somatic stem cell homeostasis

Rosario Vicidomini[1,2*], Arianna Petrizzo[1], Annamaria di Giovanni[1], Laura Cassese[1], Antonella Anna Lombardi[1], Caterina Pragliola[1] and Maria Furia[1*]

[1]Department of Biology, University of Naples "Federico II", Complesso Universitario Monte Santangelo, via Cinthia, 80126 Napoli, Italy.

[2]*Present address:* NICHD (National Institute of Child Health and Human Development)- Section on Metabolic Regulation - NIH- 35 Convent DR, Bethesda, MD 20814, USA.

***Corresponding authors:**

Maria Furia

Department of Biology, University of Naples,

via Cinthia, 80126 Napoli, Italia

Tel: +/ 39/081679072 (office); 39/081/679071 (lab)

FAX: +/ 39/081/679233

Email: mfuria@unina.it

Rosario Vicidomini

Department of Biology, University of Naples,

via Cinthia, 80126 Napoli, Italia

Tel: +/ 39/081/679071; 39/081/679074

FAX: +/ 39/081/679233

Email: rosario_vicidomini@yahoo.it

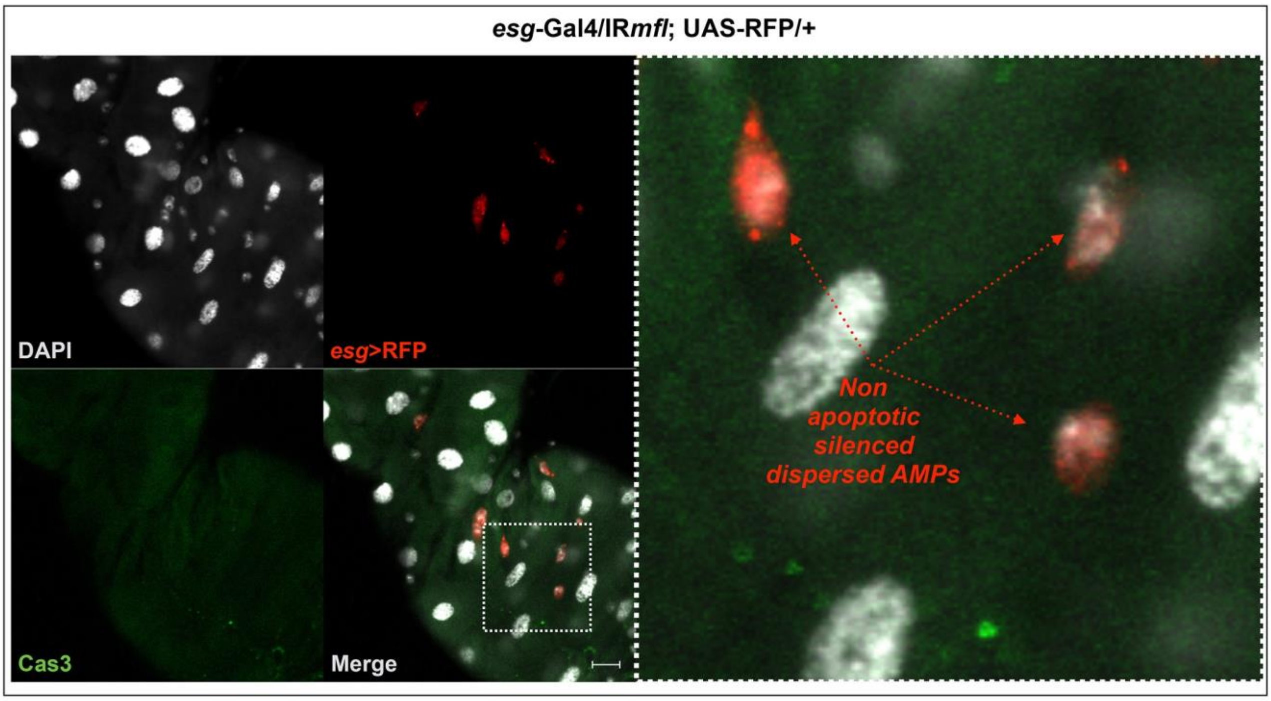

Figure S1. **Antibody against activated Cas3 fails to detect apoptotic spots in the MFL-depleted midguts.** Midguts where MFL depletion was restricted to *esg*+ cells were collected at the third larval stage, immunostained with the anti-activate Caspase 3 (Cas3) antibody and analyzed in epifluorescence. DAPI is in grey; anti-MFL antibody in green; *esg*-expression domain is marked in red by an UAS-RFP reporter line; anti-activated Caspase-3 is in green. Scale bar: 20 µm. No signal of activated Caspase-3 was detected throughout the midgut. Red arrows in the enlarged dashed square (on the right) indicate non apoptotic silenced *esg*+ cells.

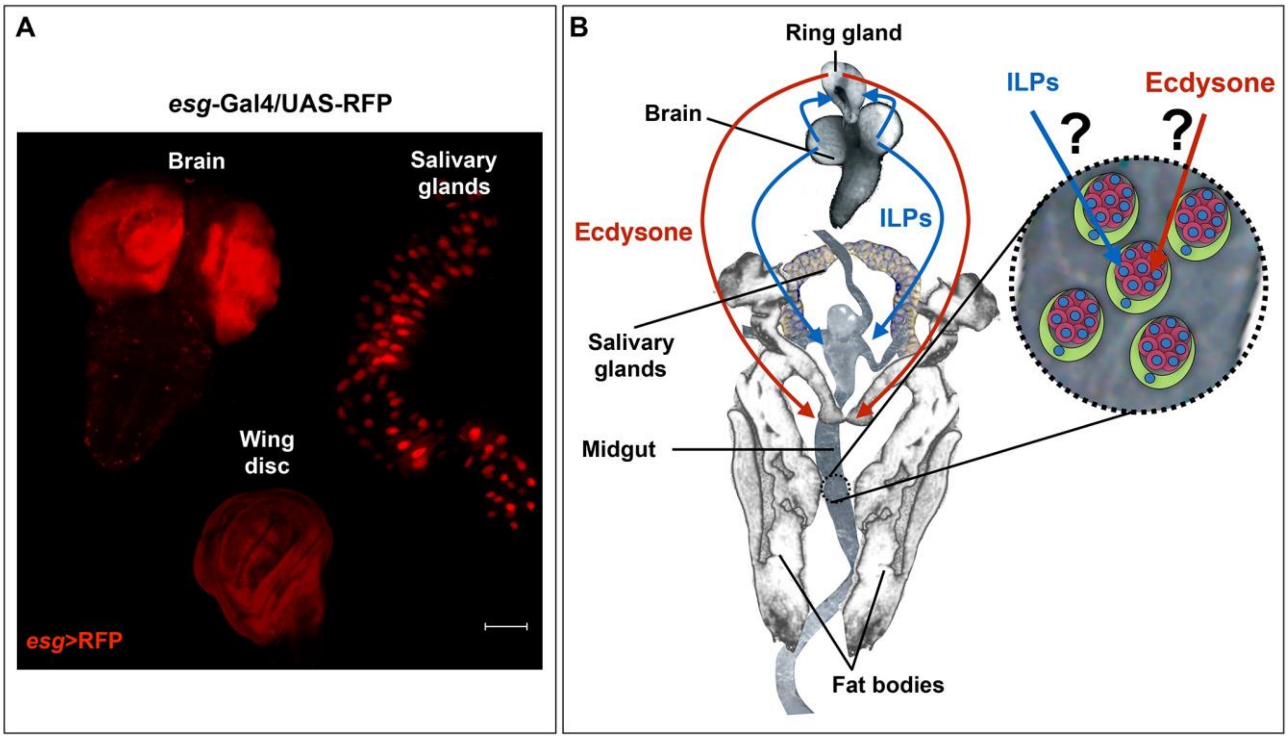

Figure S2. Overview of *esg* expression domains and related signalings that could putatively influence the formation of midgut stem cell niches. (A) Epifluorescence image of an *esg*-Gal4/UAS-RFP dissected larva. The UAS-RFP reporter marks *esg* expression compartments, which include brain, imaginal discs, and salivary glands. Scale bar: 100 µm. (B) Schematic representation of Drosophila larval organs and relative regulatory circuits. Ecdysone signaling from the ring gland and insulin-like peptide signaling from brain could potentially influence the formation of midgut imaginal islands in cell non-autonomous manner.

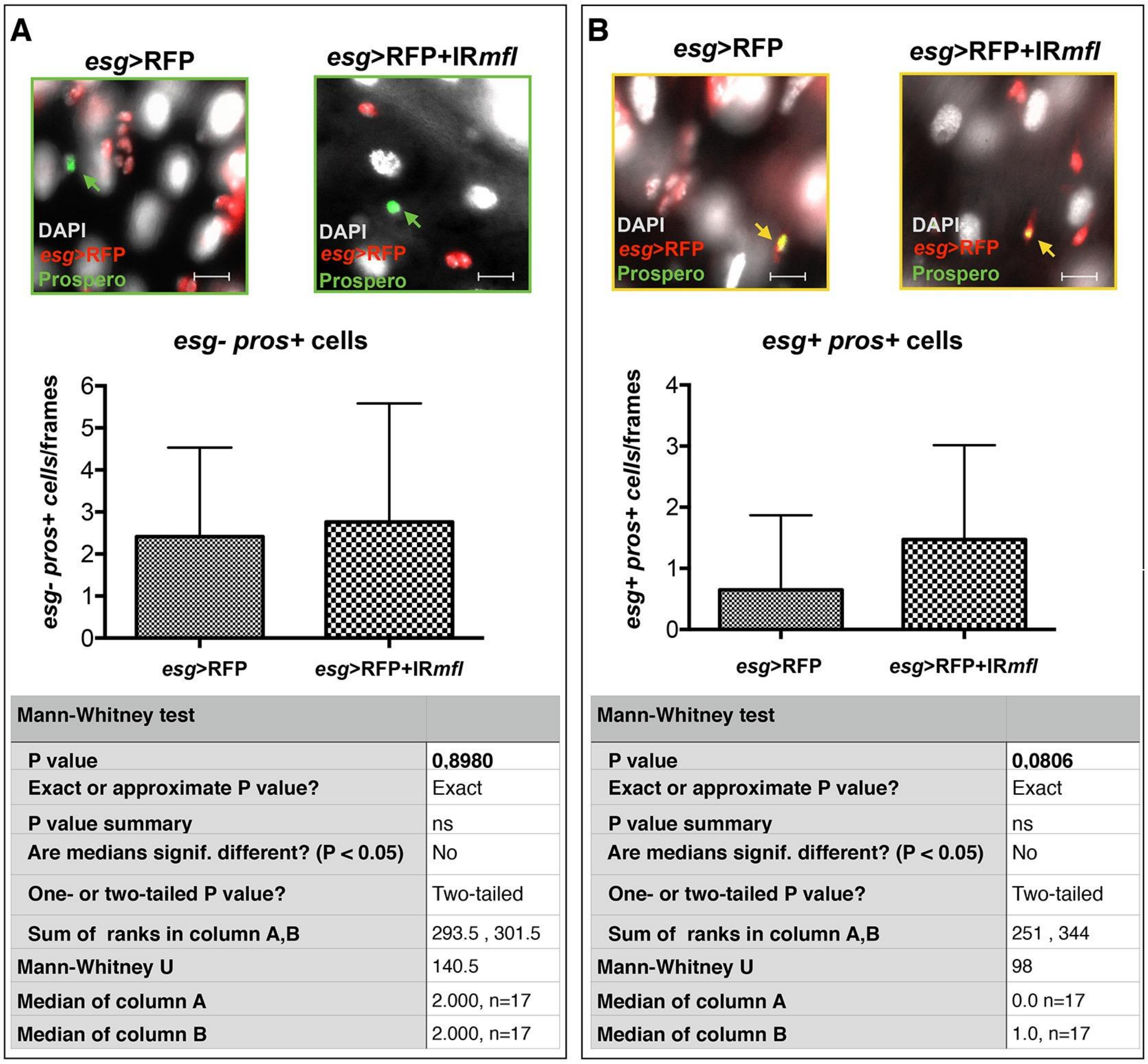

A

Mann-Whitney test	
P value	**0,8980**
Exact or approximate P value?	Exact
P value summary	ns
Are medians signif. different? (P < 0.05)	No
One- or two-tailed P value?	Two-tailed
Sum of ranks in column A,B	293.5 , 301.5
Mann-Whitney U	140.5
Median of column A	2.000, n=17
Median of column B	2.000, n=17

B

Mann-Whitney test	
P value	**0,0806**
Exact or approximate P value?	Exact
P value summary	ns
Are medians signif. different? (P < 0.05)	No
One- or two-tailed P value?	Two-tailed
Sum of ranks in column A,B	251 , 344
Mann-Whitney U	98
Median of column A	0.0 n=17
Median of column B	1.0, n=17

Figure S3. Quantification of enteroendocrine Prospero+ cells in dyskerin-depleted midguts. Control (*esg*>RFP) and silenced (*esg*>RFP+IRmfl) midguts at the third larval stage were stained with anti-Prospero antibody and counted. DAPI is in grey; *esg* expression domain is marked in red by RFP; anti-Prospero is in green. Scale bars: 20 µm. The Mann-Whitney test for variance analysis was applied to evaluate the statistical significance. According to the test, MFL depletion did not induce a significant change in the number of either the abundant *esg- pros+* (A) and the rarer *esg+ pros+* (B) enteroendocrine cells.

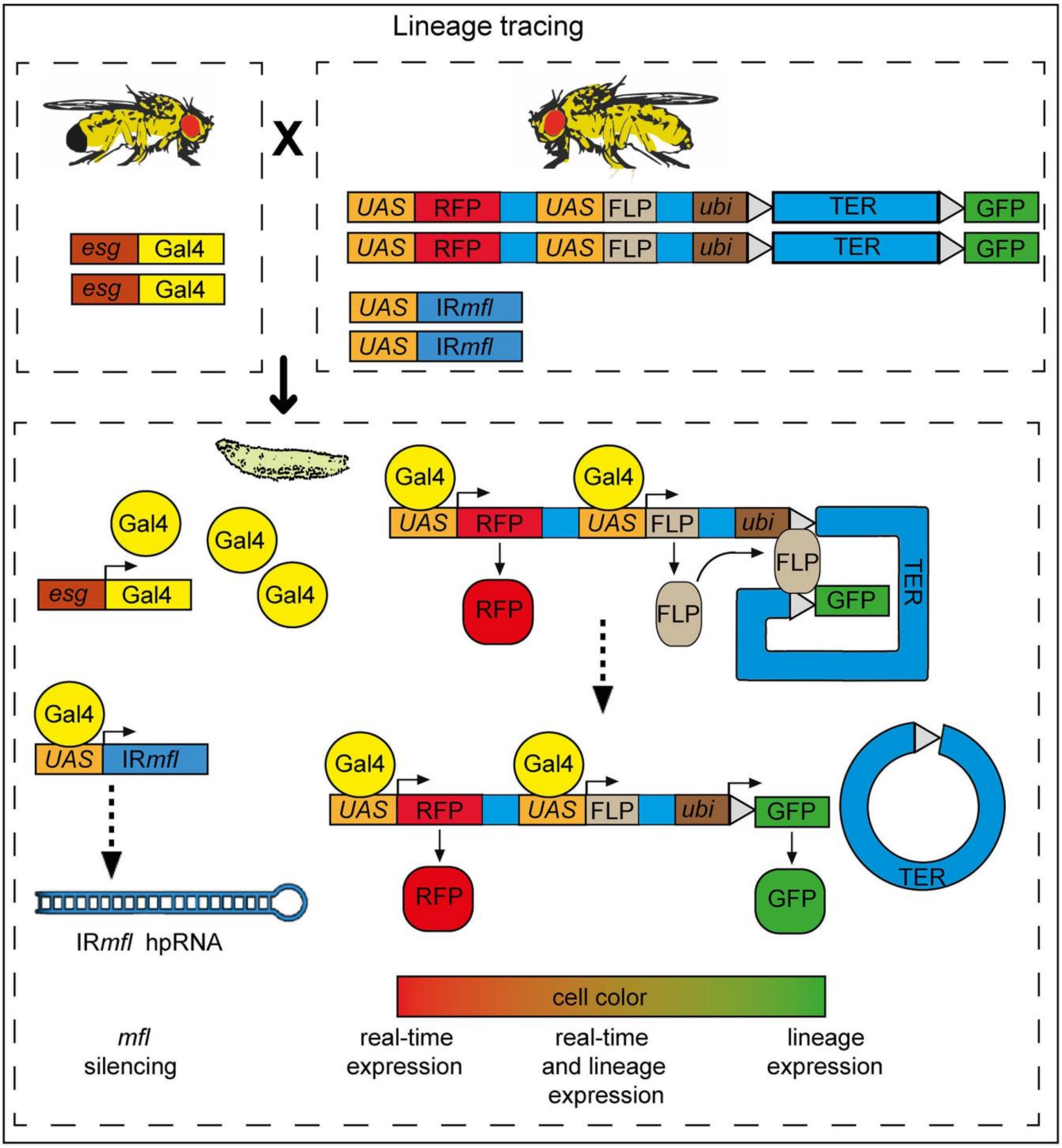

Figure S4. Schematic representation of *esg*+ cell lineage tracing strategy. Genetic crosses between the *esg*-Gal4 driver line and UAS-RFP,UAS-FLP, *ubi*>STOP>GFP/+ or UAS-IR*mfl*;UAS-RFP,UAS-FLP,*ubi*>STOP>GFP/+ responder lines generated a progeny in which Gal4 activates expression of RFP, Flippase (FLP) and IR*mfl* only in *esg*+ cells. The FLP activates the GFP coding sequence (under the control of the ubiquitin promoter) by removing an internal cassette containing a

transcriptional termination site and flanked on both sides by FRT sites. Cells which actively express *esg* are thus marked by RFP; differentiated cells deriving from *esg*+ precursors, in which FLP has been previously expressed, are marked by GFP. Cells deriving from *esg*+ precursors which continue to express *esg* are marked instead by both GFP and RFP.

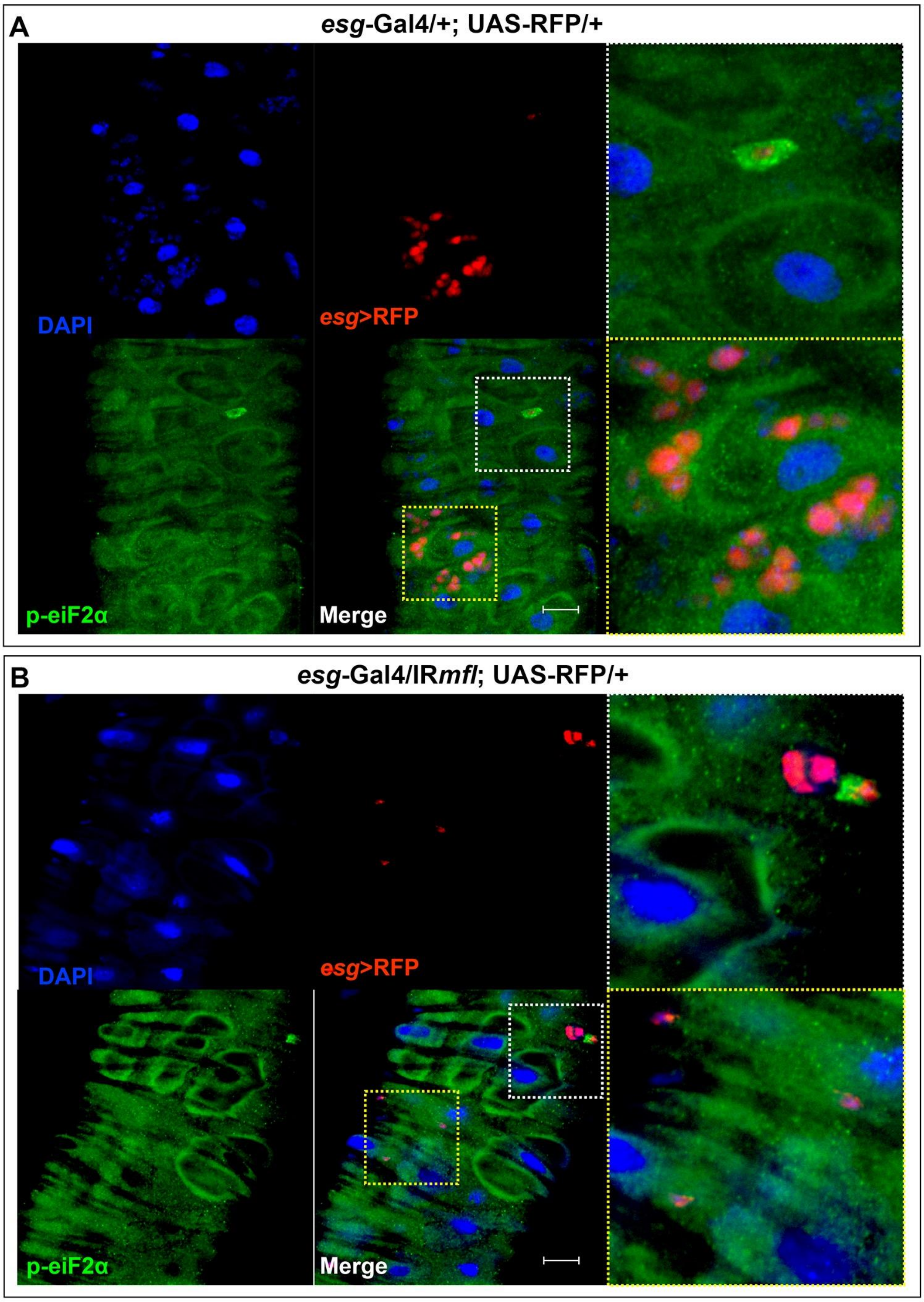

Figure S5. **Dyskerin depletion does not affect expression of p-eiF2α in midgut stem cell precursors.** Confocal images of control (A) and silenced midguts (B) after immunostaining with anti-

p-eiF2α at the third larval stage. DAPI is in blue; the *esg* expression domain is marked in red by RFP; anti-peiF2α is in green. Scale bars: 20 μm. In control midguts (A), the imaginal islands are unlabeled by anti-p-eiF2α (see the yellow dashed square and its enlargement); in contrast, *esg*+ ee cells were efficiently labeled (see white dashed square and its enlargement), thus providing an internal positive control. Similarly, in silenced midguts (B), the *esg*+ dispersed cells were unlabeled by anti-p-eiF2α (see the yellow dashed square and its enlargement), while esg+ ee cells were efficiently labeled (see white dashed square and its enlargement).
